# Twist1 induces distinct cell states depending on TGFBR1-activation

**DOI:** 10.18632/oncotarget.8878

**Published:** 2016-04-20

**Authors:** Diana Dragoi, Anja Krattenmacher, Vivek K. Mishra, Johanna M. Schmidt, Uwe J. Kloos, Lisa K. Meixner, Stefanie M. Hauck, Felix Buggenthin, Dennis Schwartz, Carsten Marr, Steven A. Johnsen, Christina H. Scheel

**Affiliations:** ^1^ Institute of Stem Cell Research, Helmholtz Center Munich, Neuherberg, Germany; ^2^ Department of General, Visceral and Pediatric Surgery, University Medical Center, Georg-August-University Göttingen, Göttingen, Germany; ^3^ Research Unit Protein Science, Helmholtz Center Munich, Neuherberg, Germany; ^4^ Institute of Computational Biology, Helmholtz Center Munich, Neuherberg, Germany

**Keywords:** Twist1, TGFBR1, epithelial-mesenchymal transition, breast cancer, context dependence

## Abstract

Basic helix-loop-helix transcription factor Twist1 is a master regulator of Epithelial-Mesenchymal Transition (EMT), a cellular program implicated in different stages of development as well as metastatic dissemination of carcinomas. Here, we show that Twist1 requires TGF-beta type-I receptor (TGFBR1)-activation to bind an enhancer region of downstream effector *ZEB1*, thereby inducing *ZEB1* transcription and EMT. When TGFBR1-phosphorylation is inhibited, Twist1 generates a distinct cell state characterized by collective invasion, simultaneous proliferation and expression of endothelial markers. By contrast, TGFBR1-activation directs Twist1 to induce stable mesenchymal transdifferentiation through EMT, thereby generating cells that display single-cell invasion, but lose their proliferative capacity. In conclusion, preventing Twist1-induced EMT by inhibiting TGFβ-signaling does not generally block acquisition of invasion, but switches mode from single-cell/non-proliferative to collective/proliferative. Together, these data reveal that transient Twist1-activation induces distinct cell states depending on signaling context and caution against the use of TGFβ-inhibitors as a therapeutic strategy to target invasiveness.

## INTRODUCTION

Twist1 orchestrates a variety of cellular programs in development and tumor progression [1, 2]. For many processes, the molecular determinants that specify the pleiotropic actions of this master regulator remain unknown. For example, Twist1 has been shown to contribute to tumor progression either by promoting collective invasion [3], generating invadopodia [4], or through induction of an Epithelial-Mesenchymal Transition (EMT) [5]. During EMT, cells lose apico-basal polarity, downregulate epithelial cell-cell adhesion molecules, and switch to a front-to-back polarity [6, 7]. Using immortalized human mammary epithelial cells (HMLE) [8], we show that signaling context determines whether Twist1 induces single-cell invasion through EMT or collective invasion in an EMT-independent manner. Specifically, TGF-beta type-I receptor (TGFBR1)-activation directs Twist1-binding to an enhancer region of downstream effector *ZEB1*, leading to its transcriptional activation. TGFBR1-activation is required for Twist1-induced mesenchymal transdifferentiation characterized by single-cell invasion and loss of proliferative capacity in 3D-collagen gels. However, when TGFBR1-phosphorylation and thus, activation is inhibited, Twist1 induces a distinct cell state characterized by collective invasion and proliferation, as well as expression of endothelial cell surface markers, such as CD31. Together, our results demonstrate that signaling context directs the outcome of Twist1-activation. To develop effective therapeutic strategies against Twist1-mediated tumor progression, epigenetic or genetic context, such as mutations in TGFβ-pathways, need to be taken into account.

## RESULTS

### Twist1 requires TGFBR1-activation for EMT-induction

**Figure 1:**
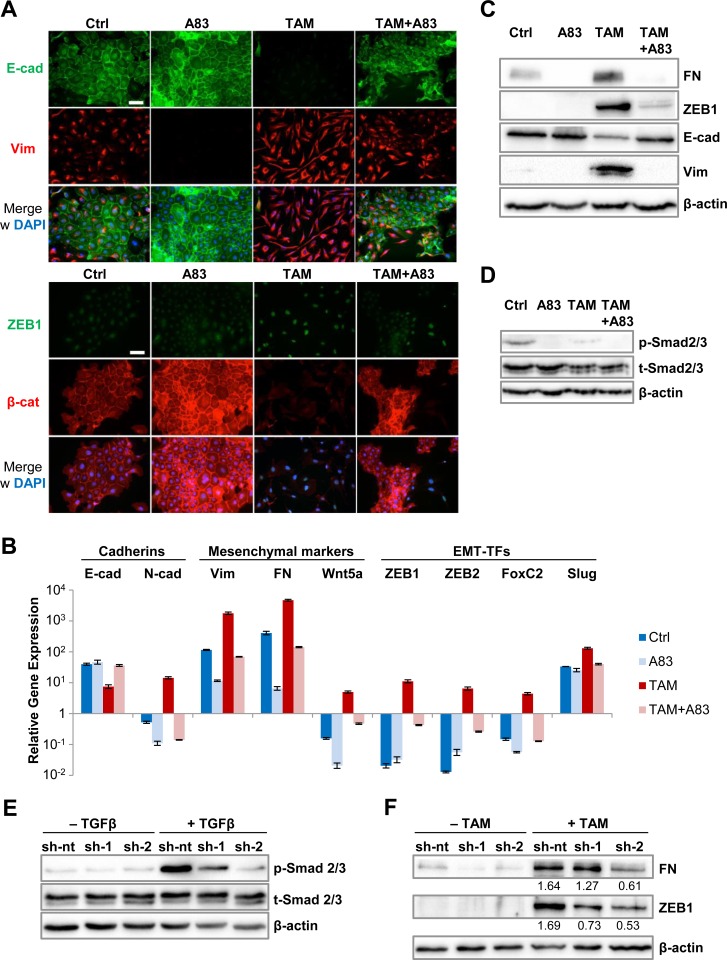
Twist1 requires TGFBR1-activation for EMT-induction **A.** Immunofluorescence: E-cadherin (green), Vimentin (red), ZEB1 (green) and β-catenin (red), 40,6-diamidino-2-phenylindol-dihydrochloride (DAPI, blue). HMLE-Twist1-ER cells at 16 days post induction (dpi), treated with TGFBR1-inhibitor A83-01 (A83), 4-Hydroxy-Tamoxifen (TAM) and TAM+A83. Control (Ctrl) = untreated. Scale bar: 100 μm. **B.** RT-PCR: E-cadherin, N-cadherin, Vimentin, Fibronectin (FN), Wnt5a, ZEB1, ZEB2, FoxC2 and Slug mRNA expression. Samples treated as described in (A). *n* = 3. **C.** Immunoblot: Fibronectin (FN), ZEB1, E-cadherin, Vimentin and β-actin. Samples treated as described in (A). **D.** Immunoblot: phosphorylated (p-), total (t-) Smad2/3 and β-actin. Samples treated as described in (A). **E.** Immunoblot: phosphorylated (p-), total (t-) Smad2/3 and β-actin. HMLE-Twist1-ER cells transduced with non-targeting control (sh-nt) or sh-RNAs targeting TGFBR1 (sh-1 or sh-2). Cells were treated with 2 ng/ml recombinant TGF-β for 45 min before lysis. **F.** Immunoblot: Fibronectin (FN), ZEB1 and β-actin. Cells generated as described in (E). Cells were treated with TAM for 8 days. Data are presented as mean ± SEM.

To determine whether Twist1 requires a specific signaling context to induce EMT, we utilized the CD24-purified, epithelial fraction of HMLE cells transduced with Twist1 coupled to a mutated Estrogen Receptor (ER)-ligand binding-domain (Figure S1A) [9]. To test whether continuous TGFβ-signaling was required for Twist1-induced EMT, a small-molecule inhibitor of TGFBR1-phosphorylation, A83-01, was applied [10]. Over a period of 16 days, 4-hydroxy-Tamoxifen (TAM) or TAM+A83-01 was added every 2 days. TAM-treated cells progressively transdifferentiated to a mesenchymal state, marked by loss of adherens junctions through downregulation of membranous E-cadherin and β-catenin, upregulation of mesenchymal intermediary filament Vimentin [11,] strong nuclear expression of the zinc-finger EMT-TF ZEB1, and acquisition of a front-to-back polarity (Figure 1A). By contrast, cells treated with TAM+A83-01 retained membranous E-cadherin and β-catenin expression and failed to upregulate Vimentin and ZEB1. Quantification of protein and mRNA by immunoblot and RT-PCR, respectively, revealed that TGFBR1-inhibition suppressed Twist1-mediated downregulation of E-cadherin, upregulation of a broad panel of mesenchymal markers, Slug and ZEB1, which are direct repressors of E-cadherin and function downstream of Twist1 (Figure 1B and 1C) [12–15]. In conclusion, the Twist1-induced EMT-transcriptional program depended on TGFBR1-activation. By immunoblot, we detected low, but robustly phosphorylated Smad2/3 protein, indicative of endogenous TGFβ-signaling in control cells, which was blocked by treatment with A83-01 (Figure 1D). These findings were supported by Smad-Binding Element (SBE)-reporter assays (Figure S1B). Twist1-activation decreased Smad2/3-phosphorylation, suggesting a negative feedback (Figure 1D). However, when TGFβ was added exogenously, SBE-reporter activity increased 6-fold in TAM-treated cells compared to a 2.5-fold in control cells, suggesting that Twist1 increases sensitivity of cells to TGFβ (Figure S1C).

In contrast to TGFβ-signaling, Twist1 did not require activation of other pathways generally implicated in EMT [2]. HMLE-Twist1-ER cells treated either with TAM+XAV939, an inhibitor of canonical Wnt signaling [16], or with TAM+JNK-inhibitor SP600125 acquired a mesenchymal phenotype (Figure S1D). These data indicate that Twist1 induces EMT independently of canonical Wnt and JNK signaling in HMLE cells. To genetically validate our findings, we performed shRNA-mediated knockdown of TGFBR1, which impaired TGFβ-induced Smad2/3 phosphorylation (Figures 1E and S1E). However, attenuation of Twist1-induced EMT was less efficient than pharmacological inhibition of TGFBR1-phosphorylation (Figures 1F and S1F). Therefore, we assessed whether other kinases with high affinity to A83-01 are required for EMT [17]. First, we tested receptor-interacting serine/threonine-protein kinase 2 (RIPK2). Its downstream target, p65 [18, 19], was phosphorylated upon Twist1-activation, but not inhibited by A83-01 (Figure S1G). Vascular growth factor receptor (VEGFR), also targeted by A83-01 [17], did not impact Twist1-induced EMT either, as shown by treating cells with Axitinib, a VEGFR2-inhibitor (Figure S1H). These data indicate that Twist1-induced EMT specifically requires TGFBR1-activation.

### TGFBR1-activation directs Twist1-binding to a *ZEB1*-enhancer region

**Figure 2:**
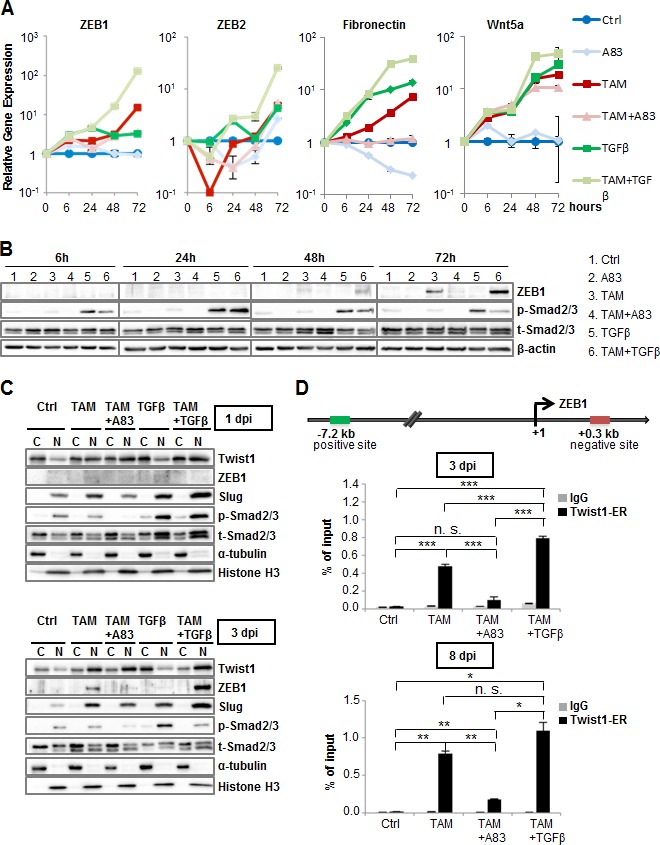
TGFBR1-activation directs Twist1-binding to a *ZEB1*-enhancer region **A.** RT-PCR: ZEB1, ZEB2, Fibronectin and Wnt5a. HMLE-Twist1-ER treated with TGFBR1-inhibitor A83-01 (A83), recombinant TGFβ, 4-Hydroxy-Tamoxifen (TAM), TAM+A83 and TAM+TGFβ, at 6, 24, 48 and 72 hours post induction (dpi). Timepoint 0 was artificially set at value 1. Control (Ctrl) = untreated. Gene expression was normalized to control cells for each timepoint. *n* = 3. **B.** Immunoblot: ZEB1, phosphorylated (p-), total (t-) Smad2/3 and β-actin. Cells treated as described in (A). **C.** Immunoblot: Twist1, ZEB1, Slug, phosphorylated (p-), total (t-) Smad2/3, α-tubulin and Histone H3 in cytoplasmic (C) and nuclear fractions (N). HMLE-Twist-ER cells treated analogous to (A) at 1 dpi or 3 dpi. **D.** Chromatin Immunoprecipitation: Twist1-binding upstream of the *ZEB1* gene in HMLE-Twist1-ER cells treated analogous to (A) at 3dpi and 8dpi. IgG was used as antibody control. *n* = 3. *p < 0.05, **p < 0.01, ***p < 0.001. Data are presented as mean ± SEM.

To elucidate how TGFBR1-activation impacts target-gene induction by Twist1, we examined transcript levels of downstream effectors and mesenchymal markers 6, 24, 48 and 72 hours after Twist1-activation. TAM-treatment resulted in robustly detectable increases of ZEB1, ZEB2, Fibronectin and Wnt5a within 24 hours (Figure 2A). Concomitant addition of TGFβ further increased ZEB1 gene expression. By contrast, cells treated with TAM+A83-01 or TGFβ alone displayed significantly lower mRNA levels of ZEB1 and fibronectin (Figure 2A). ZEB1-protein levels paralleled these findings (Figure 2B). Since ZEB1 and the miR-200 family repress each other in a negative feedback loop [20, 21], we determined whether TGFBR1-activation affected miR-200 family expression within this early time frame. However, while Twist1-activation led to a 1.5-fold downregulation of miR-200a, this effect was independent of TGFBR1-phosphorylation (Figure S2A).

Since A83-01 or TGFβ did not affect protein levels or nuclear translocation of Twist1 (Figure 2C), we hypothesized that TGFBR1-activation modulates Twist1-chromatin binding. Indeed, Chromatin Immunoprecipitation (ChIP) analysis confirmed that Twist1 binds to a DNA-sequence 7.2 kb upstream of the transcription start site of *ZEB1*, identified though a recently published ChIP-sequencing data set of Twist1 (Figures 2D and S2B) [22]. Depending on TGFBR1-activation, TAM-treatment induced Twist1-occupancy, indicating identification of an enhancer-region of *ZEB1*-transcription. However, no binding of Smad3 over a large region upstream of *ZEB1* was detectable in a previously published ChIP-sequencing data set, in sharp contrast to TGFβ-target-gene *JUNB* (Figure S2C). In conclusion, we show Twist1 binds to an enhancer-region required for *ZEB1*-transcription in a TGFBR1-dependent manner. However, while we did not exclude direct Twist1-Smad interaction, our findings indicate that Smads do not transcriptionally activate *ZEB1*.

### TGFβ accelerates Twist1-induced mesenchymal transdifferentiation

**Figure 3:**
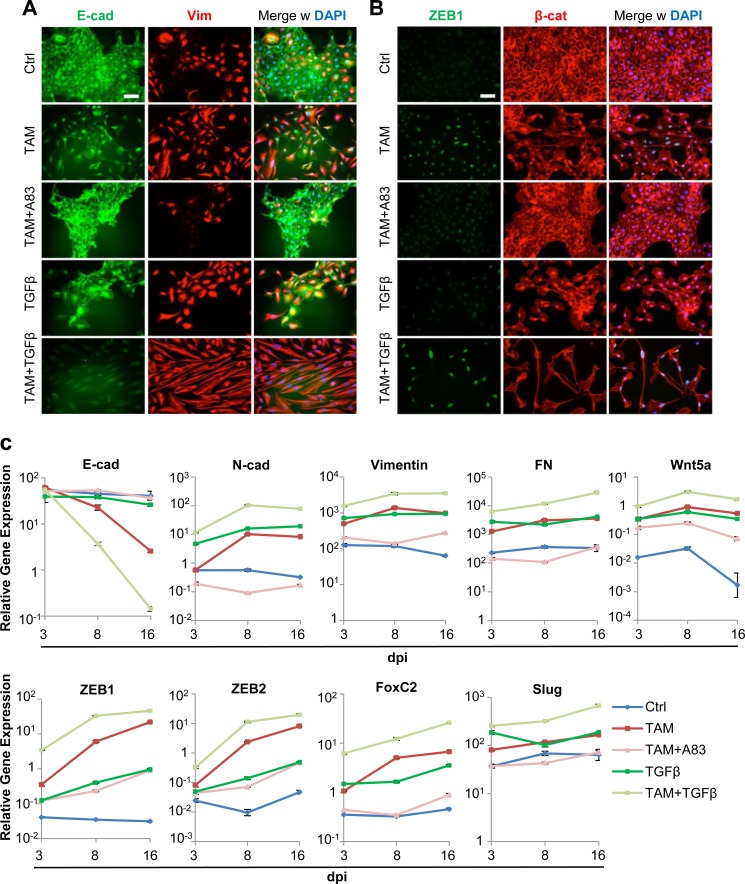
TGFβ accelerates Twist1-induced mesenchymal transdifferentiation **A.** Immunofluorescence: E-cadherin (green), Vimentin (red), DAPI (blue). HMLE-Twist1-ER treated with recombinant TGFβ, 4-Hydroxy-Tamoxifen (TAM), TAM+ TGFβ and TAM+TGFBR1-inhibitor A83-01 (A83), at 8 days post induction. Control (Ctrl) = untreated. Scale bar: 100 μm **B.** Immunofluorescence: ZEB1 (green), β-catenin (red) and DAPI (blue). Cells treated as described in (A). **C.** RT-PCR: E-cadherin, N-cadherin, Vimentin, Fibronectin (FN), Wnt5a, ZEB1, ZEB2, FoxC2 and Slug. HMLE-Twist1-ER treated with recombinant TGFβ, 4-Hydroxy-Tamoxifen (TAM), TAM+ TGFβ and TAM+TGFBR1-inhibitor A83-01 (A83), at 3 days, 8 days and 16 days post induction. Timepoint 0 was artificially set at value 1. Gene expression was normalized to control cells for each timepoint. Control (Ctrl) = untreated. *n* = 3.

Since exogenous TGFβ increased Twist1-induced ZEB1-expression, we determined whether ZEB1-levels were rate-limiting for EMT-induction. Indeed, cells treated with TAM+TGFβ transitioned to a mesenchymal state within 8 days compared to TAM (Figures 3A, 3B and 1A). Cells treated with TAM or TGFβ had undergone a partial EMT, based on the persistence of epithelial colonies (Figure 3A and 3B). Moreover, TGFβ did not upregulate ZEB1 expression (Figure 3B), indicating that TGFβ contributes to loss of adherens junctions, but requires Twist1-activation to induce ZEB1 and therefore, transcriptional repression of E-cadherin. Transcript levels of E-cadherin, mesenchymal markers and Twist1-downstream effectors supported these conclusions (Figure 3C).

Given the implications of EMT in many different tissues and cancer types [2], we set out to test whether combining exogenous TGFβ treatments with activation of Twist1 also boosts the EMT process in other cellular systems than the human breast. We therefore transduced the human lung carcinoma cell line A549 with the Twist1-ER construct (A549-Twist1-ER). In contrast to the results obtained in HMLE-Twist1-ER cells, we observed that TGFβ treatment alone was sufficient for the transcriptional downregulation of E-cadherin expression in A549-Twist1-ER cells (Figure S3A). However, in concordance with our previous observations, activating Twist1 by TAM in addition to TGFβ treatment further increased the transcriptional level of the mesenchymal markers fibronectin and Wnt5a, and significantly enhanced expression of EMT-TFs, such as ZEB1, ZEB2 and Slug compared to cells treated only with TGFβ (Figure S3A). This suggests that Twist1 and TGFβ signaling cooperate to induce EMT and that the underlying mechanism is not restricted to the human breast.

To investigate whether Twist1 requires TGFβ signaling not only for induction, but also for maintenance of the mesenchymal phenotype, we continued treating HMLE-Twist1-ER cells with TAM beyond the 16 days required for EMT induction and added A83-01 for another 6 days (Figure S3B). Under these conditions, we observed that A83-01 did not cause reversion to an epithelial state, thereby suggesting that Twist1 induces irreversible changes in cell state, resulting in loss of TGFβ-dependency. Indeed, we could confirm this hypothesis by showing that the transcriptional profiles before and after TAM withdrawal were very similar (Figure S3C). This indicated that once HMLE-Twist1-ER cells had reached a mesenchymal state, continuous Twist1-activity was no longer required to maintain it. We hypothesized cell-state stability was due to disruption of the miR-200-ZEB1 feedback-loop. Indeed, all miR-200 family members were significantly downregulated in TAM-treated cells, but not in those continuously treated with TAM+A83-01 (Figure S3D). Together, our data indicated that TGFβ-signaling controls the ability of Twist1 to induce ZEB1 and thereby, mesenchymal transdifferentiation.

### Twist1 induces invasive proliferation independently of TGFBR1-activation

**Figure 4:**
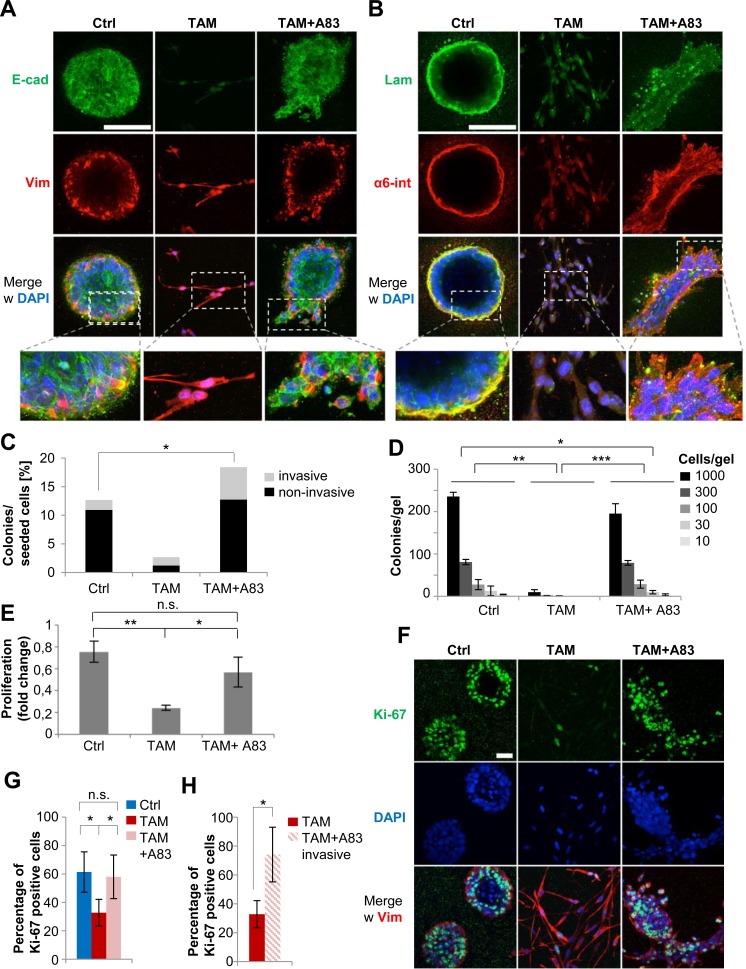
Twist1 induces invasive proliferation independently of TGFBR1-activation **A.** Immunofluorescence: E-cadherin (green), Vimentin (red), DAPI (blue). HMLE-Twist1-ER cells treated with 4-Hydroxy-Tamoxifen (TAM), TGFBR1-inhibitor A83-01 (A83) and TAM+A83 for 16 days, then seeded into into 3D-collagen gels and cultured for 8 days without further treatment. Control (Ctrl) = untreated. Scale bar: 100 μm. **B.** Immunofluorescence: Laminin (green), α6-integrin (red), DAPI (blue). Cells treated as described in (A). Scale bar: 100 μm. **C.** Quantification of invasive and non-invasive structures based on (A), (B). Each multicellular structure displaying at least one multicellular protrusion was considered invasive. Number of colonies as percentage of seeded cells. Colony-count for each condition was: *n* = 190 for Ctrl, *n* = 40 for TAM and *n* = 276 for TAM+A83. *p < 0.05. **D.** 3D-collagen gels: quantification of colonies plated at indicated densities. Cells treated as described in (A). *n* = 3. **p < 0.01, ***p < 0.001. **E.** 3D-collagen gels: quantification of proliferation by cell-count relative to the seeding density per gel. Cells treated as in (A). *n* = 3. *p < 0.05, **p < 0.01. **F.** Immunofluorescence: Ki-67 (green) and Vimentin (red), DAPI (blue). Cells treated and cultured as described in (A). Scale bar: 100 μm. **G.** Quantification of Ki-67-positive cells as shown in (F). Cells counted for each condition: *n* = 1732 for Ctrl, *n* = 640 for TAM and *n* = 1043 for TAM+A83. ***p < 0.001. **H.** Quantification of Ki-67-positive cells within invasive protrusions in TAM+A83 condition. Cells counted for each condition: *n* = 640 for TAM and *n* = 175 for TAM+A83 invasive. *p < 0.05. Data are presented as mean ± SEM.

Next, we determined whether TGFBR1-activation was required for Twist1-induced migration and invasion. Continuous treatment with TAM or TAM+A83-01 for 16 days resulted in a stable transcriptional profile that persisted after drug-withdrawal (Figure S3C). Therefore, cells were seeded without further stimulation into 3-dimensional (3D)-collagen gels, an assay previously used to demonstrate that Twist1-activation induces single-cell invasion [9]. Consistent with their non-invasive phenotype, the majority of control cells generated multicellular spheres with membranous E-cadherin, basal localization of Vimentin and deposition of the basement membrane-component Laminin, co-localized with its binding-partner α6-integrin (Figure 4A and 4B). As expected, TAM-treated cells displayed a mesenchymal phenotype, maintained nuclear ZEB1 expression and were attenuated in colony-formation (Figures 4A-4D and S4A) and proliferation (Figure 4E). By contrast, TAM+A83-01-treated cells generated invasive multicellular structures with membranous E-cadherin, but diffuse localization of Laminin and α6-integrin (Figures 4A-4C and S4A). F-actin-staining revealed an abundance of filopodia-like protrusions, underscoring the invasive phenotype (Figure S4B) [23]. Together, these data demonstrated that transient Twist1-activation induces sustained invasion. Importantly, the mode of invasion induced by Twist1 depended on TGFBR1; single-cell invasion was induced by Twist1 when TGFBR1 was active, collective invasion when TGFBR1-phosphorylation was blocked. Importantly, TGFBR1-independent motility was revealed in a 3D-environment that mimics physical properties of the human mammary gland [24]. By contrast, migration assays and single-cell tracking conducted in standard 2D-conditions suggested that TGFBR1-inhibition completely blocked Twist1-induced motility (Figures S4C-S4E).

Since mesenchymal cells were strongly growth-inhibited in 3D-collagen gels (Figure 4C-4E), we assessed proliferation specifically of the actively invading cells generated by TAM+A83-01. As expected, Ki-67 staining revealed approximately 50% fewer Ki-67-positive cells in TAM-treated compared to control or TAM+A83-01-treated cells (Figure 4F and 4G). Remarkably, the percentage of Ki-67-positive cells within the invasive multicellular protrusions of TAM+A8-01 cells was 2- to 3-fold higher than in TAM-treated cells, suggesting they were not growth-inhibited, despite actively invading (Figure 4H). Importantly, the anti-proliferative effects of Twist1 were not detectable in standard 2D-cell culture conditions: single-cell tracking revealed only minor delays in cell-cycle duration and, consequently, proliferation rates (Figures S4F and S4G). These observations suggested that standard 2D-cell culture masked anti-proliferative effects resulting from Twist1-activation. In conclusion, inhibition of TGFBR1-activity re-directed Twist1 to generate cells that invade and proliferate simultaneously, a trait that was revealed in 3D-collagen gels, but not in 2D-cell culture.

### Twist1 induces endothelial cell surface proteins depending on TGFBR1-inhibition

**Figure 5:**
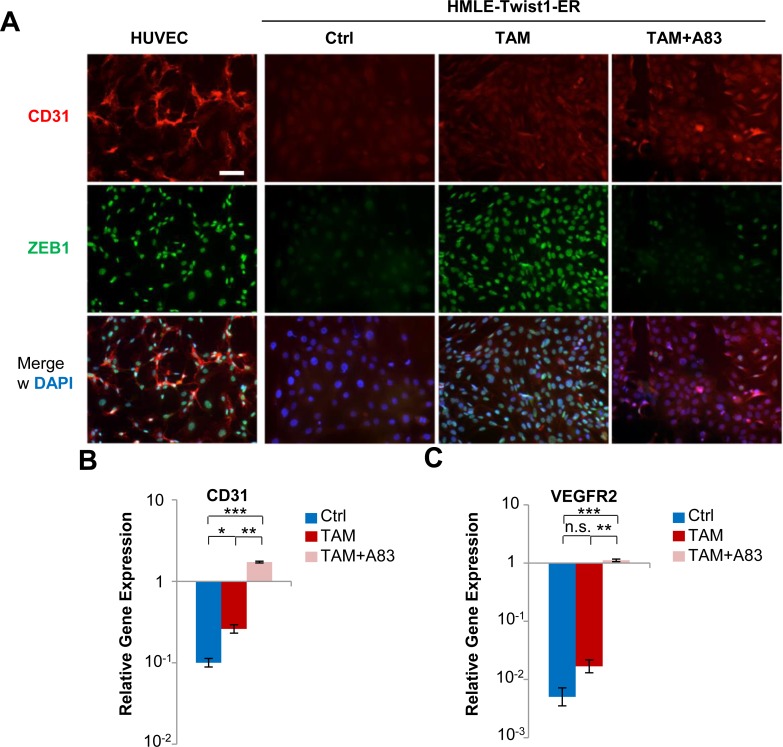
Twist1 induces endothelial cell surface proteins depending on TGFBR1-inhibition **A.** Immunofluorescence: CD31 (red), ZEB1 (green), DAPI (blue). HMLE-Twist1-ER cells treated with 4-Hydroxy-Tamoxifen (TAM), TGFBR1-inhibitor A83-01 (A83) and TAM+A83 for 16 days, then plated without further treatment and fixed after 3 days culture. Control (Ctrl) = untreated. HUVEC cells were used as a positive control. Scale bar: 100 μm. **B.** RT-PCR: CD31. Cells treated as in (A). *n* = 3. **p < 0.01. **C.** RT-PCR: VEGFR2. Cells treated as in (A). *n* = 3. **p < 0.01. Data are presented as mean ± SEM.

To identify cell surface markers specifically overexpressed in invasive 3D-structures generated by TAM+A83-01-treated cells, we performed cell-surface enriching proteomics. Thus, we identified 125 cell surface markers expressed at least at 2-fold higher levels in 3D-structures generated by cells treated with TAM+A83-01 compared to controls (Table S1). Among the top-10 upregulated proteins, we identified endothelial adhesion molecules CD31 and CD99. We validated CD31-expression by immunofluorescence in TAM- or TAM+A83-01-treated cells (Figure 5A). At the transcriptional level, CD31 was 20-fold upregulated in TAM+A83-01- compared to TAM-treated and control cells (Figure 5B). In line with these results, Twist1 has been shown to promote vasculogenic mimicry in breast cancer [25]. Consistently, transcript levels of the angiogenic receptor VEGFR2 were strongly induced in TAM+A83-01-treated cells, but nearly undetectable in TAM-treated or control cells (Figure 5C). In conclusion, depending on TGFBR1-activation, Twist1-activation in HMLE cells induces either EMT or a unique cell state characterized by collectively invading, proliferative cells that express endothelial proteins.

## DISCUSSION

Twist1 is correlated with tumor progression, because it was found to be upregulated in breast cancer and its expression has been associated with poor survival [26–28]. Consistently, Twist1 has been shown to promote breast cancer metastasis to the lung [5]. However, continuous Twist1 activation was revealed to inhibit proliferation of disseminated tumor cells at distant sites, whereas transient expression of Twist1 re-enabled colonization and resulted in metastatic outgrowth [9, 29]. Our study offers insights into context-depending effects of Twist1 that may reconcile some of these these apparently contradicting results. Accordingly, we propose that Twist1-activation may lead to different outcomes in tumors depending on whether TGFβ-signaling is functional or disabled. More precisely, we observe that TGF B R1-activation directs Twist1 to bind an enhancer region required for *ZEB1*-transcription. Since ZEB1 represses the miR-200 family and E-cadherin [20, 21], our observations suggest that the decision whether a cell undergoes an EMT or retains epithelial cell-identity depends on the ability of Twist1 to induce ZEB1, which in turn, depends on TGFβ-signaling. Thereby, our results add to a growing number of studies showing that Twist1-activation needs to be evaluated in context of signal-duration [9, 29], dosage [30, 31] and pathway-activation [32, 33].

At the functional level, TGFβ-signaling directs Twist1 to generate singly migrating and invading cells with greatly attenuated proliferative capacity. In the absence of TGFβ-signaling, Twist1 induces cells that collectively invade while proliferating simultaneously. These data suggest that Twist1 can promote metastatic progression independently of EMT, since collectively invading cells that retain proliferative capacity might harbor increased metastatic potential. This hypothesis is supported by the observation that circulating clusters of epithelial tumor cells in the bloodstream are rare, but much more potent in giving rise to actively growing metastases [34]. Moreover, inactivating mutations of TGFβ-pathway-members occur frequently in breast cancer and are associated with an increased metastatic potential [35–37]. Since TGFβ acts as a tumor suppressor by inhibiting cell proliferation [38], tumors overexpressing Twist1 and harboring inactivating mutations of TGFβ-pathway-members are highly likely to overcome growth inhibition, while being able to collectively invade. Along the same lines, our results suggest that patients suffering from breast tumors overexpressing Twist1 may not profit from therapies targeting TGFβ pathway components.

When TGFβ-signaling is inhibited, Twist1-actions are redirected to generate a distinct cell surface profile, characterized by expression of endothelial proteins such as CD31, CD99 and VEGFR2. Future studies should address whether these proteins promote metastatic outgrowth, for example by stimulating angiogenesis.

## MATERIALS AND METHODS

### Cell lines

Human mammary epithelial cells (HMLE) were generated as previously described [39]. HMLE cells transduced with the pWZL-Twist1-ER plasmid [14] were referred to as HMLE-Twist1-ER cells and propagated in mammary epithelial growth medium (PromoCell) containing 5% Pen/Strep (Invitrogen) and blasticidin (10 μg/ml, Sigma). A549 cells transduced with the pWZL-Twist1-ER were referred to as A549-Twist1-ER cells and propagated in F12/K medium (Gibco Life Technologies) containing 10% FCS (Pan Biotech), 5% Pen/Strep (Invitrogen) and blasticidin (10 μg/ml, Sigma). For EMT induction, cells were treated with 4-hydroxytamoxifen (TAM, 20 nM in 100% Ethanol, Sigma). Human recombinant TGFβ1 was used at a concentration of 2 ng/ml (in 4 mM HCl containing 1mg/ml bovine serum albumin, R&D Systems). Small-molecule inhibitor experiments were performed using A83-01 (1 μM, Tocris), XAV-939 (1 μM, Enzo Life Sciences), SP600125 (200 nM, Tocris) and Axitinib (50 nM, Tocris), dissolved in DMSO (Sigma). Medium and drugs/ TGFβ1 were changed every 48h, except for short time course experiments (every 24h). HEK293T cells were cultured in DMEM medium (Gibco Life Technologies), supplemented with 10% FCS (Pan Biotech) and 5% Pen/Strep (Invitrogen). HUVEC cells were cultered in M-199 HBS medium (Amimed) mixed 1:1 with Endothelial Cell Growth Medium Kit classic (Pelo Biotech) and supplemented with 10% FCS (Pan Biotech) and 5% Pen/Strep (Invitrogen).

### Culture in floating 3D-collagen gels

Floating 3D-collagen gels were prepared as previously described (Linnemann et al., 2015). Briefly, single-cell suspensions were mixed with neutralization solution and rat-tail collagen I (BD Biosciences) at a final collagen-concentration of 1.3 mg/ml. The gel mixture was poured into siloxane-coated 24-well plates and allowed to polymerize at 37°C for 1h. Gels were then detached from the well, media was added and gels were kept in culture for the indicated number of days. Growth medium including drugs was changed every 2 days. For proliferation measurements, gels were digested with Collagenase I (Sigma) followed by trypsinization to obtain a single-cell suspension that was counted and normalized to initial seeding density.

### Knockdown of TGFBR1

pGIPZ vectors expressing shRNAs targeting TGFBR1 or a non-targeting control (nt) together with GFP were purchased from GE Healthcare (# RHS4346; sh-1, clone ID V2LHS_55964; sh-2, clone ID V3LHS_305780). For virus production, ca. 2×10^6^ HEK293T cells were transfected with pGIPZ vectors, pCMV-dR8.2 dvpr, pCMV-VSV-G and X-tremeGENE HP DNA Transfection Reagent according to manufacturer's instructions (Roche). Fluorescence-Activated Cell Sorting (FACS) was used to select GFP-positive cells.

### Statistical analysis

Data are presented as mean ±SEM. A Student's test (two-tailed) was used to compare two groups, where *p* < 0.05 was considered significant, unless otherwise indicated.

## SUPPLEMENTARY MATERIALS FIGURES AND TABLE



## References

[1] Nieto MA (2011). The ins and outs of the epithelial to mesenchymal transition in health and disease. Annu Rev Cell Dev Biol.

[2] Thiery JP, Acloque H, Huang RYJ, Nieto MA (2009). Epithelial-mesenchymal transitions in development and disease. Cell.

[3] Shamir ER, Pappalardo E, Jorgens DM, Coutinho K, Tsai WT, Aziz K, Auer M, Tran PT, Bader JS, Ewald AJ (2014). Twist1-induced dissemination preserves epithelial identity and requires E-cadherin. J Cell Biol.

[4] Eckert MA, Lwin TM, Chang AT, Kim J, Danis E, Ohno-Machado L, Yang J (2011). Twist1-induced invadopodia formation promotes tumor metastasis. Cancer Cell.

[5] Yang J, Mani SA, Donaher JL, Ramaswamy S, Itzykson RA, Come C, Savagner P, Gitelman I, Richardson A, Weinberg RA (2004). Twist, a master regulator of morphogenesis, plays an essential role in tumor metastasis. Cell.

[6] Hay ED, Zuk A (1995). Transformations between epithelium and mesenchyme: normal, pathological, and experimentally induced. Am J Kidney Dis.

[7] Lamouille S, Xu J, Derynck R (2014). Molecular mechanisms of epithelial-mesenchymal transition. Nat Rev Mol Cell Biol.

[8] Elenbaas B, Spirio L, Koerner F, Fleming MD, Zimonjic DB, Donaher JL, Popescu NC, Hahn WC, Weinberg RA (2001). Human breast cancer cells generated by oncogenic transformation of primary mammary epithelial cells. Genes Dev.

[9] Schmidt JM, Panzilius E, Bartsch HS, Irmler M, Beckers J, Kari V, Linnemann JR, Dragoi D, Hirschi B, Kloos UJ, Sass S, Theis F, Kahlert S (2015). Stem-cell-like properties and epithelial plasticity arise as stable traits after transient Twist1 activation. Cell Rep.

[10] Tojo M, Hamashima Y, Hanyu A, Kajimoto T, Saitoh M, Miyazono K, Node M, Imamura T (2005). The ALK-5 inhibitor A-83-01 inhibits Smad signaling and epithelial-to-mesenchymal transition by transforming growth factor-beta. Cancer Science.

[11] Savagner P (2010). The epithelial-mesenchymal transition (EMT) phenomenon. Ann Oncol.

[12] Eger A, Aigner K, Sonderegger S, Dampier B, Oehler S, Schreiber M, Berx G, Cano A, Beug H, Foisner R (2005). DeltaEF1 is a transcriptional repressor of E-cadherin and regulates epithelial plasticity in breast cancer cells. Oncogene.

[13] Alves CC, Carneiro F, Hoefler H, Becker K-F (2009). Role of the epithelial-mesenchymal transition regulator Slug in primary human cancers. Front Biosci (Landmark Ed).

[14] Casas E, Kim J, Bendesky A, Ohno-Machado L, Wolfe CJ, Yang J (2011). Snail2 is an essential mediator of Twist1-induced epithelial mesenchymal transition and metastasis. Cancer Res.

[15] Dave N, Guaita-Esteruelas S, Gutarra S, Frias À, Beltran M, Peiró S, de Herreros AG (2011). Functional cooperation between Snail1 and twist in the regulation of ZEB1 expression during epithelial to mesenchymal transition. J Biol Chem.

[16] Huang SM, Mishina YM, Liu S, Cheung A, Stegmeier F, Michaud GA, Charlat O, Wiellette E, Zhang Y, Wiessner S, Hild M, Shi X, Wilson CJ (2009). Tankyrase inhibition stabilizes axin and antagonizes Wnt signalling. Nature.

[17] Vogt J, Traynor R, Sapkota GP (2011). The specificities of small molecule inhibitors of the TGFβ and BMP pathways. Cellular Signalling.

[18] Meylan E, Tschopp J (2005). The RIP kinases: crucial integrators of cellular stress. Trends Biochem Sci.

[19] Zhang D, Lin J, Han J (2010). Receptor-interacting protein (RIP) kinase family. Cell Mol Immunol.

[20] Burk U, Schubert J, Wellner U, Schmalhofer O, Vincan E, Spaderna S, Brabletz T (2008). A reciprocal repression between ZEB1 and members of the miR-200 family promotes EMT and invasion in cancer cells. EMBO Rep.

[21] Bracken CP, Gregory PA, Kolesnikoff N, Bert AG, Wang J, Shannon MF, Goodall GJ (2008). A double-negative feedback loop between ZEB1-SIP1 and the microRNA-200 family regulates epithelial-mesenchymal transition. Cancer Res.

[22] Chang AT, Liu Y, Ayyanathan K, Benner C, Jiang Y, Prokop JW, Paz H, Wang D, Li HR, Fu XD, Rauscher FJ, Yang J (2015). An evolutionarily conserved DNA architecture determines target specificity of the TWIST family bHLH transcription factors. Genes Dev.

[23] Shibue T, Brooks MW, Weinberg RA (2013). An integrin-linked machinery of cytoskeletal regulation that enables experimental tumor initiation and metastatic colonization. Cancer Cell.

[24] Linnemann JR, Miura H, Meixner LK, Irmler M, Kloos UJ, Hirschi B, Bartsch HS, Sass S, Beckers J, Theis FJ, Gabka C, Sotlar K, Scheel CH (2015). Quantification of regenerative potential in primary human mammary epithelial cells. Development.

[25] Zhang D, Sun B, Zhao X, Ma Y, Ji R, Gu Q, Dong X, Li J, Liu F, Jia X, Leng X, Zhang C, Sun R (2014). Twist1 expression induced by sunitinib accelerates tumor cell vasculogenic mimicry by increasing the population of CD133+ cells in triple-negative breast cancer. Molecular Cancer.

[26] Martin TA, Goyal A, Watkins G, Jiang WG (2005). Expression of the transcription factors snail, slug, and twist and their clinical significance in human breast cancer. Ann Surg Oncol.

[27] Watanabe O, Imamura H, Shimizu T, Kinoshita J, Okabe T, Hirano A, Yoshimatsu K, Konno S, Aiba M, Ogawa K (2004). Expression of twist and wnt in human breast cancer. Anticancer Res.

[28] Soini Y, Tuhkanen H, Sironen R, Virtanen I, Kataja V, Auvinen P, Mannermaa A, Kosma VM (2011). Transcription factors zeb1, twist and snai1 in breast carcinoma. BMC Cancer.

[29] Tsai JH, Donaher JL, Murphy DA, Chau S, Yang J (2012). Spatiotemporal regulation of epithelial-mesenchymal transition is essential for squamous cell carcinoma metastasis. Cancer Cell.

[30] Beck B, Lapouge G, Rorive S, Drogat B, Desaedelaere K, Delafaille S, Dubois C, Salmon I, Willekens K, Marine JC, Blanpain C (2015). Different levels of Twist1 regulate skin tumor initiation, stemness, and progression. Cell Stem Cell.

[31] Sharma VP, Fenwick AL, Brockop MS, McGowan SJ, Goos JA, Hoogeboom AJ, Brady AF, Jeelani NO, Lynch SA, Mulliken JB, Murray DJ, Phipps JM, Sweeney E (2013). Mutations in TCF12, encoding a basic helix-loop-helix partner of TWIST1, are a frequent cause of coronal craniosynostosis. Nat Genet.

[32] Connerney J, Andreeva V, Leshem Y, Mercado MA, Dowell K, Yang X, Lindner V, Friesel RE, Spicer DB (2008). Twist1 homodimers enhance FGF responsiveness of the cranial sutures and promote suture closure. Dev Biol.

[33] Lee MP, Ratner N, Yutzey KE (2014). Genome-wide Twist1 occupancy in endocardial cushion cells, embryonic limb buds, and peripheral nerve sheath tumor cells. BMC Genomics.

[34] Aceto N, Bardia A, Miyamoto DT, Donaldson MC, Wittner BS, Spencer JA, Yu M, Pely A, Engstrom A, Zhu H, Brannigan BW, Kapur R, Stott SL (2014). Circulating tumor cell clusters are oligoclonal precursors of breast cancer metastasis. Cell.

[35] Chen T, Carter D, Garrigue-Antar L, Reiss M (1998). Transforming growth factor beta type I receptor kinase mutant associated with metastatic breast cancer. Cancer Res.

[36] Chen T, Jackson CR, Link A, Markey MP, Colligan BM, Douglass LE, Pemberton JO, Deddens JA, Graff JR, Carter JH (2006). Int7G24A variant of transforming growth factor-beta receptor type I is associated with invasive breast cancer. Clinical Cancer Research.

[37] Yang L, Moses HL (2008). Transforming growth factor beta: tumor suppressor or promoter? Are host immune cells the answer?. Cancer Res.

[38] Massagué J (2008). TGFbeta in Cancer. Cell.

[39] Elenbaas B, Weinberg RA (2001). Heterotypic signaling between epithelial tumor cells and fibroblasts in carcinoma formation. Experimental Cell Research.

