# Supplementary material for: Twist1 induces distinct cell states depending on TGFBR1-activation
Source: Oncotarget. 2016 Apr 20;7(21):30396–407. doi: 10.18632/oncotarget.8878 (PMC5058688; doi:10.18632/oncotarget.8878)
Supplement: Supplementary file 1 [file oncotarget-07-30396-s001.pdf]

Twist1 induces distinct cell states depending on TGFBR1-activation

Supplemental Materials and Methods

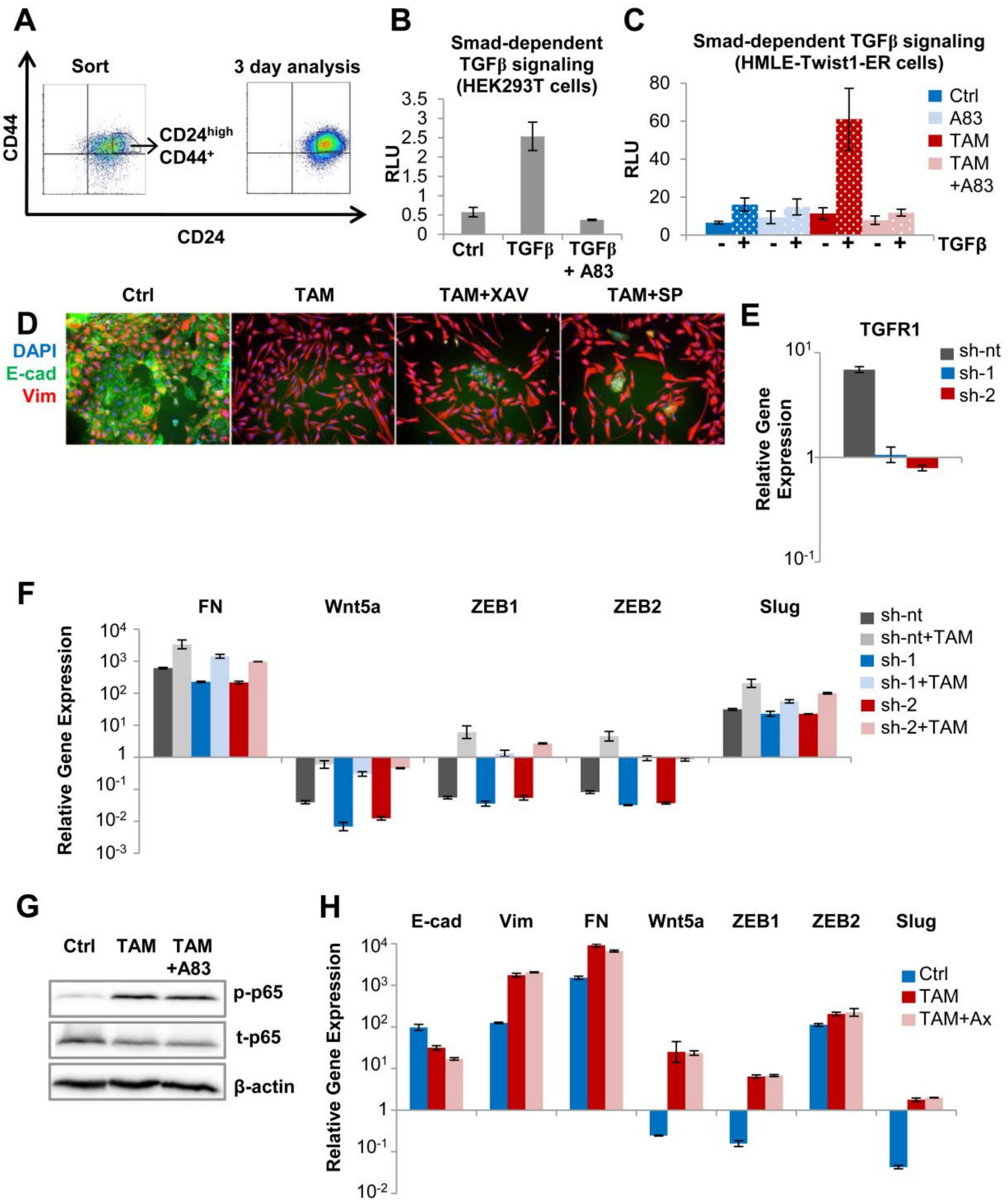

Figure S1. Twist1 requires TGFBR1-activation for EMT-induction (related to Figure 1)

(A) Fluorescent Activated Cell Sorting (FACS): CD44 and CD24 cell-surface staining and postsort analysis after 3 days in culture.

(B) Reporter assay: HEK293T cells were transfected with a reporter plasmid harboring smad binding elements (SBE) and a TGFβ1-expressing plasmid, and treated with TGFBR1-inhibitor A83-01 (A83). Reporter activity was measured after 24h. n=6.

(C) Reporter assay: HMLE-Twist1-ER cells treated with A83-01, 4-hydroxy Tamoxifen (TAM) and TAM+A83 for 16 days prior transfection with a reporter plasmid harboring smad binding elements (SBE). Recombinant TGFβ1 was added 24 hours after transfection and reporter activity was measured after another 24h. n=6.

(D) Immunofluorescence: E-cadherin (green), Vimentin (red), DAPI (blue). HMLE-Twist1-ER cells treated for 16 days with TAM, TAM+XAV939 (XAV, canonical Wnt signaling-inhibitor), TAM+SP600125 (SP, JNK-inhibitor). Scale bar: 200 µm.

(E) RT-PCR: TGFBR1. HMLE-Twist-ER cells HMLE-Twist1-ER cells transduced with non-targeting control (sh-nt) or sh-RNAs targeting TGFBR1 (sh-1 or sh-2). n=3.

(F) RT-PCR: Fibronectin (FN), Wnt5a, ZEB1, ZEB2, Slug. Cells as described in (E), 8 days post-induction with TAM. n=3

(G) Immunoblot: phosphorylated (p-) p65, total (t-) p65, β-actin. HMLE-Twist1-ER were treated for 16 days with TAM or TAM+A83-01 (A83).

(H) RT-PCR: E-cadherin, Vimentin, Fibronectin (FN), ZEB1, ZEB2 and Slug. HMLE-Twist1-ER cells treated with TAM or TAM+Axitinib (Ax) for 16 days. n=3.

Data are presented as mean ± SEM.

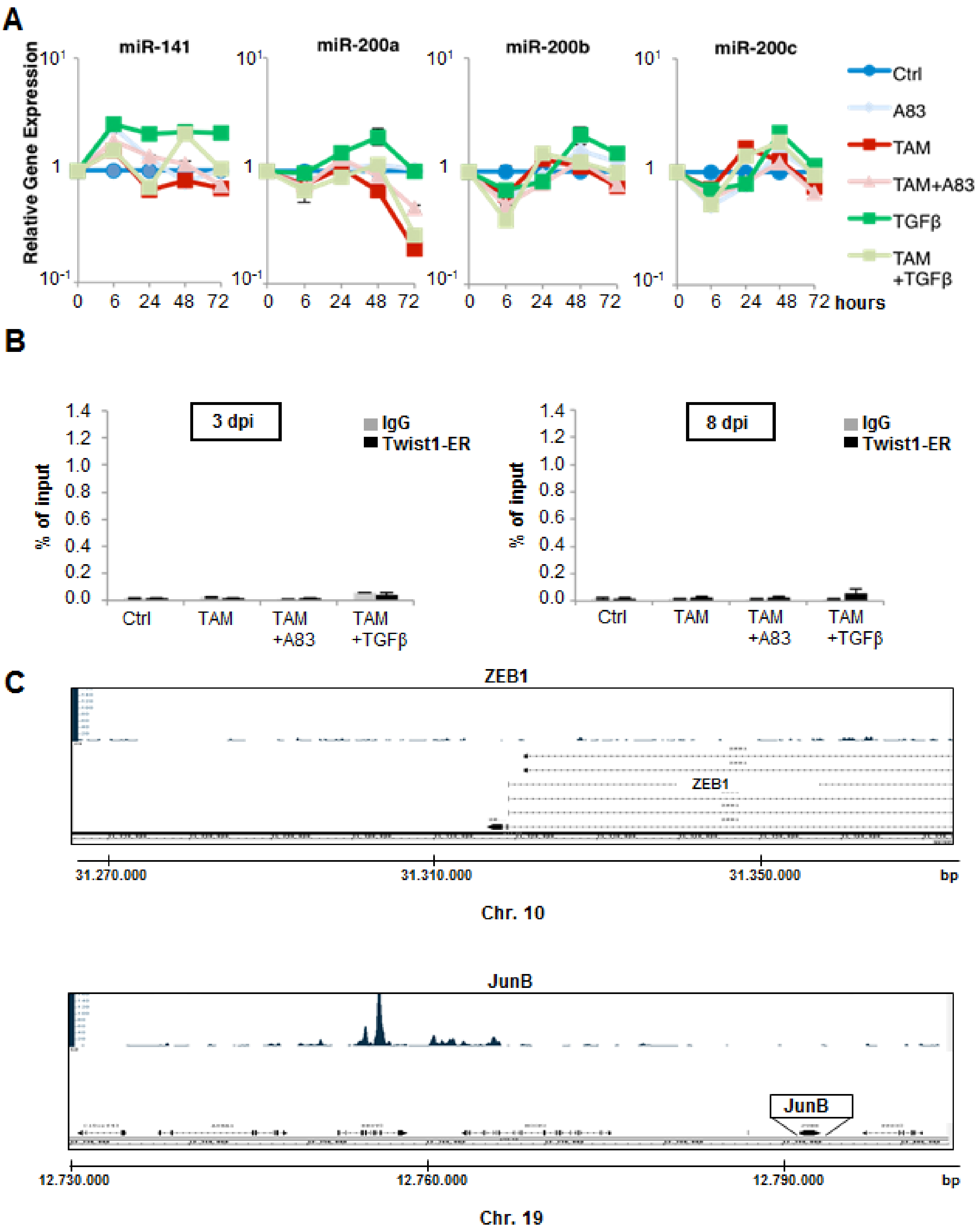
A
miR-141
miR-200a
miR-200b
miR-200c
Relative Gene Expression
10^1
1
10^{-1}
0 6 24 48 72
hours
Ctrl
A83
TAM
TAM+A83
TGFβ
TAM +TGFβ
B
3 dpi
8 dpi
IgG
Twist1-ER
% of input
1.4 1.2 1.0 0.8 0.6 0.4 0.2 0.0
Ctrl
TAM
TAM +A83
TAM +TGFβ
C
ZEB1
31.270.000
31.310.000
31.350.000
bp
Chr. 10
JunB
12.730.000
12.760.000
12.790.000
bp
Chr. 19

Figure S2. TGFBR1-activation directs Twist1-binding to a ZEB1-enhancer region (related to Fig. 2)

(A) RT-PCR: miR-141, miR-200a, miR-200b and miR-200c. HMLE-Twist1-ER treated with TGFBR1-inhibitor A83-01 (A83), recombinant TGFβ, 4-Hydroxy-Tamoxifen (TAM), TAM+A83 and TAM+TGFβ, at 6, 24, 48 and 72 hours post induction (dpi). Timepoint 0 was artificially set at value 1. Control (Ctrl) = untreated. miR expression was normalized to control cells for each timepoint. n=3.

(B) Chromatin Immunoprecipitation: Twist1-binding within the coding region of the *ZEB1* gene (= Negative site, refer to Figure 2D) in HMLE-Twist1-ER cells treated analogous to (A) at 3dpi and 8dpi. IgG was used as antibody control. n=3.

(C) Chromatin Immunoprecipitation-sequencing, imported from data set GSM934616, visualized with IGB (http://bioviz.org/igb/index.html): Smad3-binding upstream and within the coding region of *ZEB1* and *JUNB*.

Data are presented as mean ± SEM.

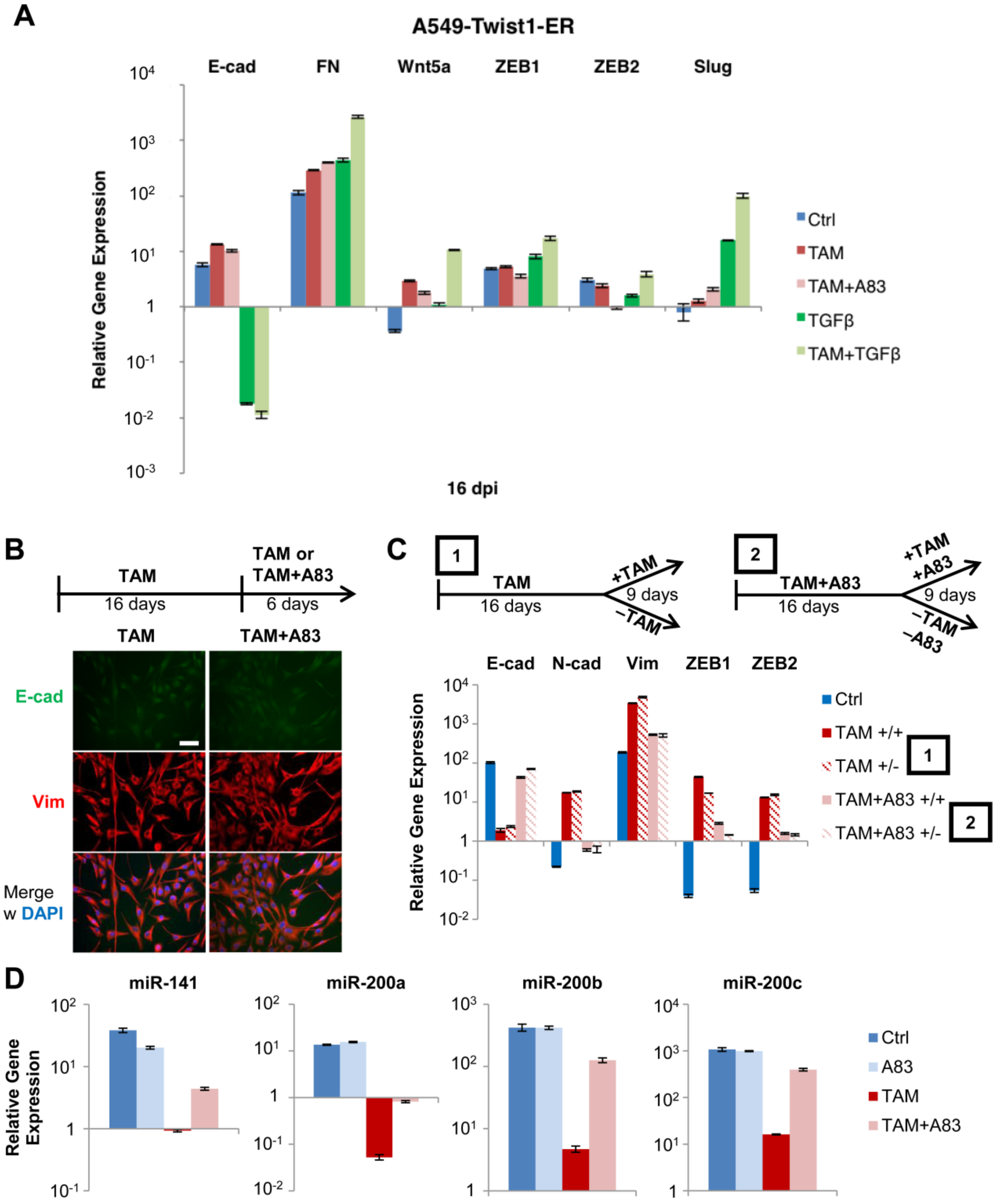

Figure S3. TGFβ accelerates Twist1-induced EMT (related to Figure 3)

(A) RT-PCR: E-cadherin, Fibronectin (FN), Wnt5a, ZEB1, ZEB2, Slug. A549-Twist1-ER cells treated with recombinant TGFβ, 4-Hydroxy-Tamoxifen (TAM), TAM+TGFβ and

TAM+TGFR1-inhibitor A83-01 (A83), at 16 days post induction (dpi). Control (Ctrl) = untreated. n=3.

(B) Immunofluorescence: E-cadherin (green), Vimentin (red), DAPI (blue). HMLE-Twist1-ER cells treated with TAM for 16 days, followed by treatment with TAM+TGFBR1-inhibitor A83-01 (A83) for 6 days. Scale bar: 100 µm.

(C) RT-PCR: E-cadherin, N-cadherin, Vimentin, ZEB1 and ZEB2. HMLE-Twist1-ER cells treated with TAM for 16 days, followed by treatment with TAM+A83-01 (A83) with controls as indicated. n=3.

(D) RT-PCR: miR-141, miR-200a, miR-200b and miR-200c. HMLE-Twist1-ER cells were treated for 16 days with A83-01 (A83), TAM and TAM+A83. Control (Ctrl) = untreated. n=3.

Data are presented as mean ± SEM.

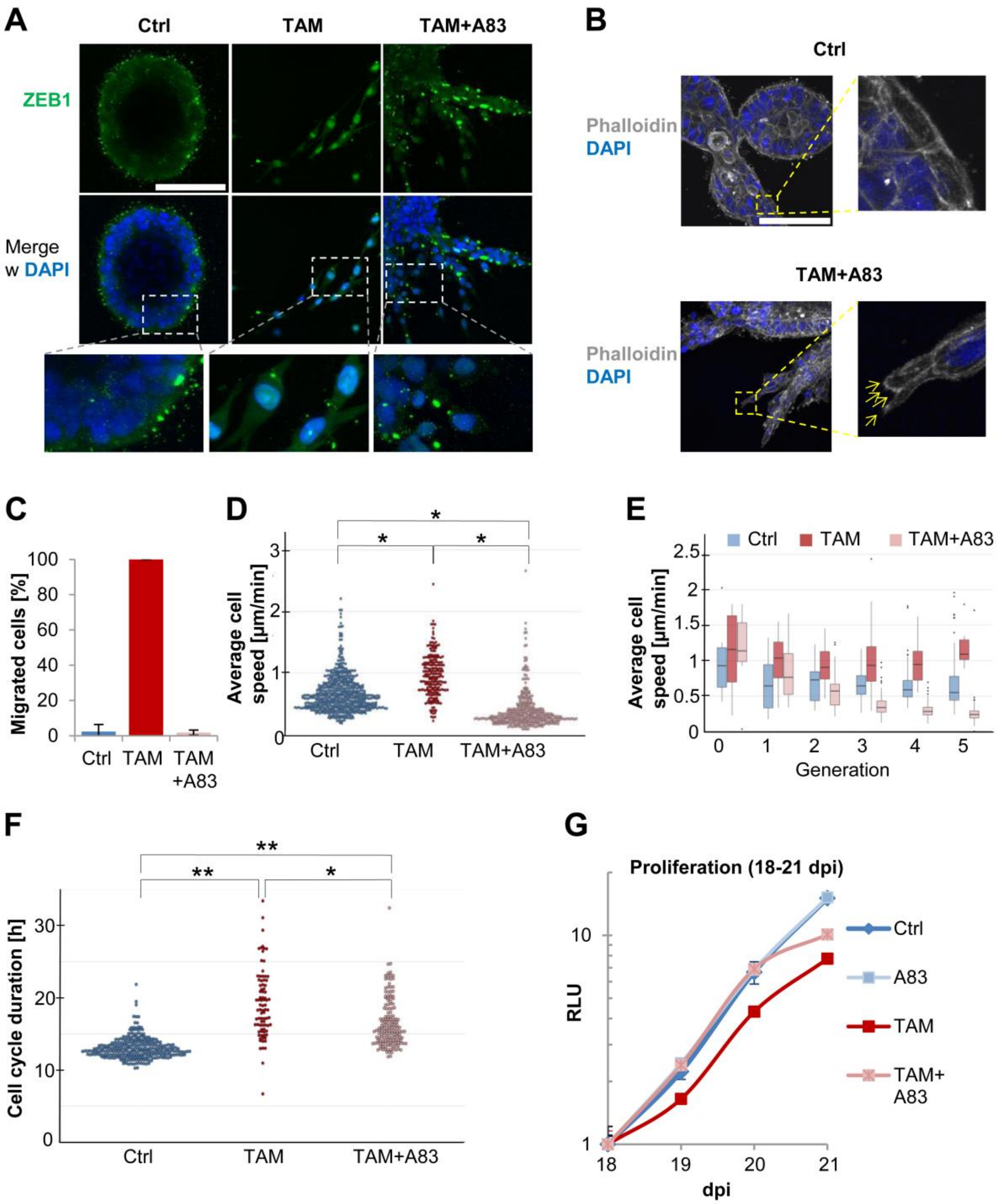

Figure 4. Twist1 induces collective invasion independently of TGFBR1-activation (related to Figure 4)

(A) Immunofluorescence: ZEB1 (green), DAPI (blue). HMLE-Twist1-ER cells treated with 4-Hydroxy-Tamoxifen (TAM), TGFBR1-inhibitor A83-01 (A83) and TAM+A83

for 16 days, then seeded into into 3D-collagen gels and cultured for 8 days without further treatment. Control (Ctrl) = untreated. Scale bar: 100 µm.

(B) Immunofluorescence: F-actin (Phalloidin, white), DAPI (blue). Cells treated as described in (A). Scale bar: 100 µm.

(C) Migration assay: quantification of migrated HMLE-Twist1-ER cells treated as described in (A). The number of cells migrated in the TAM-condition was set as 100%. n=3.

(D) Live cell imaging: quantification of average cell speed. Cells were treated as described in (A). Single cells were tracked between 10 and 13 dpi. n=563 cells for Ctrl, n=210 cells for TAM, n=338 cells for TAM+A83. One representative experiment from 3 biological replicates is shown. *p<0.001.

(E) Live cell imaging: quantification of average cell speed over five generations. Cells were treated as described in (A), tracked and quantified as described in (D). One representative experiment from 3 biological replicates is shown.

(F) Live cell imaging: quantification of cell-cycle duration (= duplication time). Cells were treated as described in (A) and tracked as described in (D). n=274 cells for Ctrl, n=84 cells for TAM, n=158 cells for TAM+A83. One representative experiment from 3 biological replicates is shown. *p<0.01, **p<0.001.

(G) Proliferation Assay: HMLE-Twist1-ER cells treated with A83-01 (A83), TAM+A83 for 21 days. Control (Ctrl) = untreated. Proliferation was measured between 18 and 21 days post induction. n=6.

Data are presented as mean ± SEM.

Supplemental Experimental Procedures

Fluorescence-Activated Cell Sorting and Flow Cytometry

Single cell suspensions were suspended in 0.1% BSA/PBS on ice prior to FACS. Cells were stained with the following antibodies: APC Mouse Anti-Human CD44 (clone G44-26; BD Biosciences: #559942) and FITC Mouse Anti-Human CD24 (clone ML5; BD Biosciences: #555427). Dead cells were excluded by 7-AAD (BD Biosciences). Cells were sorted and analyzed on a BD FACS AriaI, followed by data processing with FlowJoV10 software.

Reporter Assay

HEK293T cells were transfected with SBE4-luc [1] using X-tremeGENE HP DNA Transfection Reagent (Roche). $1x10^4$ cells were transfected with 90 ng reporter plasmid and 10 ng pGL-SV40-Renilla luciferase control plasmid. Reporter activities were measured using the Dual-Luciferase Reporter Assay System (Promega).

Migration Assay

$2.5x10^4$ cells were plated in 24-well culture inserts with 8 μm pores (BD Falcon). After 24 hours, non-migrated cells were removed from the upper side of the insert. For visualization, migrated cells were stained with the Hemacolor Rapid staining Set (Merck) according to the manufacturer’s instructions.

Proliferation Assay

3000 cells per well were seeded in white polystyrene 96-well plates. After 24h, drugs were added every 24h for a total duration of 3 days. Viability of cells was measured every 24h using Cell Titer Glo (Promega) according to the manufacturer's instructions.

Live cell imaging

$1x10^4$ cells per well were seeded in 24-well plates. After 24h, cells were imaged every 5min at 10x magnification with an Axio Observer.Z1 microscope using in-house software over a period of 3 days.

Cell tracking and data analysis

Individual cells were tracked manually using Timm's Tracking Tool (TTT) [2,3]. Each cell was tracked until either one of the following events occurred: (i) division (resulting in two new cells), (ii) apoptosis, (iii) cell loss (i.e. the tracked cell left the observable area) or (iv) the end of the imaging period has been reached. For cell cycle duration computation, only cells that were observed from origin (division of its parent) to division were considered. The cell cycle time is the time between these two events. For the calculation of cell speed, the displacement between two consecutive observations was calculated as Euclidian distance and divided by the time between two observations (5 mins). Average cell speed represents the mean displacement of a cell over all observations. This measure was calculated for all tracked cells. For statistical analysis a Kruskal-Willis test with post-hoc pairwise comparisons using Nemenyi-test: $p<001$ was employed.

RNA Preparation and RT-PCR Analysis

cDNA: mRNA was isolated using the mRNeasy Mini Kit (Qiagen), cDNA synthesis was performed with EasyScriptPlus Kit and Power SYBR Green-PCR Master Mix (Applied Biosystems) was used for PCR. Transcript levels of RPL32 were used as a control for normalization.

Micro-RNAs (miRNA): The miRNeasy Mini Kit (Qiagen) was used to isolate total RNA, reverse transcription of miRNAs was performed with the miScript RT Kit (Qiagen). PCR was performed using Power SYBR Green-PCR Master Mix (Applied Biosystems) and miScript Primer Assays (control/normalization = HS-RNU6-2_11, miR-141 = HS-miR-141_1, miR-200a = HS-miR-200a_1, miR-200b = HS-miR-200b_3, miR-200c = HS-miR200c_1, Qiagen).

Samples were run on a QuantStudio 12K Flex qPCR system (Life Technologies). Analysis was described previously [4].

Primers used for RT-PCR:

Gene	Forward	Reverse
CD31	AACAGTGTTGACATGAAGAGCC	TGTAAAACAGCACGTCATCCTT
E-cadherin	TGCCCAGAAAATGAAAAAGG	GTGTATGTGGCAATGCGTTC
Fibronectin	CAGTGGGAGACCTCGAGAAG	TCCCTCGGAACATCAGAAAC
FoxC2	GCCTAAGGACCTGGTGAAGC	TTGACGAAGCACTCGTTGAG
N-cadherin	ACAGTGGCCACCTACAAAGG	CCGAGATGGGGTTGATAATG
RPL32	CAGGGTTCGTAGAAGATTCAAGGG	CTTGGAGGAAACATTGTGAGCGATC

Slug	GGGGAGAAGCCTTTTTCTTG	TCCTCATGTTTGTGCAGGAG
TGFBR1	ACGGCGTTACAGTGTTTCTG	GCACATACAAACGGCCTATCTC
VEGFR2	CAAGACAGGAAGACCAAGAAAAGAC	GGTGCCACACGCTCTAGGA
Vimentin	GAGAACTTTGCCGTTGAAGC	GCTTCCTGTAGGTGGCAATC
Wnt5a	ATGGCTGGAAGTGCAATGTCT	ATACCTAGCGACCACCAAGAA
ZEB1	GCACAAGAAGAGCCACAAGTAG	GCAAGACAAGTTCAAGGGTTC
ZEB2	TTCCTGGGCTACGACCATAC	TGTGCTCCATCAAGCAATTC

Chromatin Immunoprecipitation (ChIP)

ChIP analysis was performed as described [5] with minor modifications. Briefly, cells were cross-linked with 1% formaldehyde for 20 min at room temperature and quenched with glycine. Following nuclei isolation with nuclear preparation buffer, these were sonicated in equal volumes of sonication buffers I and II using Bioruptor Plus (Diagenode SA). Next, chromatin extracts were cleared by centrifugation, diluted, pre-cleared with 50% sepharose 4B (Sigma-Aldrich, USA) slurry, and used for immune-precipitation with 1 µg of anti-ERα antibody (HC20) (Santa Cruz: # sc-543) or IgG control antibody (Abcam: # ab37415). The immune complexes were pulled down using blocked protein A sepharose beads and the ChIP immune complexes were washed twice with IP buffer, wash buffer and with 1X TE buffer. The washed ChIP immune complexes and inputs (10%) were treated with RNase A (0.2 µg/µl) at 37°C and reverse cross-linked with Proteinase K (20 µg/µl, Invitrogen) overnight at 65°C. The phenol-chloroform-isoamyl alcohol extraction method was used to purify DNA, followed by precipitation with LiCl and linear polyacrylamide. The precipitated DNA was dissolved in nuclease free water and amplified by PCR. PCR-reaction was performed as previously described [6]. Briefly, the reaction was carried out in 14 µl of PCR mix, 1.5 µl of 5 µM primers, 1 µl of purified DNA and 8.5 µl of dH_2O. Input DNA was used for standard curves and normalization.

Primers used for ChIP analysis:

Gene	Forward	Reverse

ZEB1-positive site	GCAGAGGCCATCATTCCACAA	TTGCAAAATCTGGCAAACACTATCA
ZEB1-negative site	TTCCATATTGAGCTGTTGCCG	AAAGCGAACAGCTCTTTCCGA

Western Blot Analysis

Whole protein lysates were prepared using RIPA buffer. Nuclear and cytoplasmic cell fractionations were done using Tween 20 lysis buffer. Protein lysates were resolved on a 10-12% SDS gel, transferred to PVDF membranes and probed with primary antibodies: E-cadherin (clone EP700Y; Biozol: #GTX61329), Fibronectin (BD Biosciences: #610078), NF-κB p65 (clone D14E12; Cell Signaling: #8242), Phospho-NF-κB p65 (Ser536) (clone 93H1; Cell signaling: #3033), Phospho-Smad2 (Ser465/467)/Smad3 (Ser423/425) (clone D27F4; Cell signaling: #8828), Slug (clone C19G7; Cell signaling: # 9585), Smad 2/3 (clone D7G7; Cell signaling: #8685), Twist1 (clone Twist2C1a; Santa Cruz: #sc-81417), Vimentin (clone D21H3; Cell signaling: #5741) and ZEB1 (clone H-102; Santa Cruz: #sc-25388). β-actin (Abcam: # ab6276), α-tubulin (clone B-5-1-2; Sigma: #T5168) and Histone H3 (abcam: # ab1791) were used as internal controls. Following incubation with HRP-linked secondary antibodies (Jackson Immunoresearch: #111-036-045 and #115-036-062) proteins were visualized with ECL reagent (GE Healthcare). Blots were visualized using the ChemiDoc System (Bio-Rad). Acquired images quantified using ImageJ software to calculate the signal intensity based on area and pixel density. Relative values were determined by subtracting the background from the lane of interest, followed by normalization to the loading control

Immunofluorescence

Cells were cultured on poly-D-lysine (Sigma) coated coverslips or in 3D collagen gels, fixed with 4% paraformaldehyde and permeabilized with 0.2% Triton X-100/PBS. After blocking with 10% goat serum (Biozol) in 0.1% BSA (Sigma), coverslips/gels were incubated overnight with primary antibodies in 0.1% BSA as following: α6-integrin (clone GOH3; Santa Cruz: #sc-19622), β-catenin (Clone 14; BD Biosciencens: #610153), CD31 (clone WM59; BioLegend: #303102), E-cadherin (clone EP700Y; Biozol: #GTX61329), E-cadherin-Alexa 488 (clone 24E10; New England Biolabs: #3199), Laminin (clone L9393; Sigma-Aldrich: #L9393), Ki-67 (Abcam: #ab15580), Phalloidin–Atto 647N (Sigma: #65906), Vimentin (clone V9; Abnova: #MAB3578) and ZEB1 (H-102; Santa Cruz: #sc-25388). Secondary antibodies were goat-anti-rabbit, -rat and -mouse coupled to Alexa 488 or -594 (Life Technologies). Cell nuclei were visualized with 40,6-diamidino-2-phenylindol-dihydrochloride (DAPI, Sigma). Slides were mounted with Aqua-Poly/Mount reagent (Polysciences).

Proteomics analysis

Glycosyl residues on intact cells were labelled with aminooxybiotin under mild oxidative conditions as described before [7,8] and after cell lysis, glycosylated cell surface proteins were enriched with streptavidin beads (IBA). After stringent washing steps, proteins were on-bead proteolysed with trypsin, followed by deglycosylation with PNGase F (NEB). Eluted peptides were combined, acidified and directly used for analysis on a LTQ-OrbitrapXL connected with an Ultimate3000 nano HPLC system

(Thermo Fisher Scientific) as described [9]. The full-scan MS spectra were acquired in the Orbitrap with a resolution of 60,000 and up to 10 most abundant peptide ions were selected for fragmentation in the linear ion trap. Peptides were identified and quantified using the Progenesis QI software (Nonlinear, Waters) and the Mascot search algorithm with the Ensembl Human public database as described [7-9].

Table S1: Cell surface proteomics analysis (related to Figure 5).

	3D			2D		
	Ratio TAM+A83/Ctrl	Ctrl	TAM+A83	Ctrl	TAM	TAM+A83
ENPP1	28.8	8	229	0	112728	1388
PCDH7	26.2	72	1874	5689	3821	663
**CD31**	**10.3**	**3620**	**37155**	**4952**	**3377**	**46122**
CNTNAP2	7.0	3006	20941	5022	0	51168
SMAGP	6.0	6928	41460	2931	56365	57863
PNP	5.1	248	1264	12309	3222	5178
CD99	4.9	1711	8322	19251	33107	87071
IGSF8	4.7	1520	7074	32350	25862	67722
ACAT2	4.3	201	868	16221	18152	14736
SLC3A2	4.2	10316	43463	126433	16686	85000
SLC1A4	4.2	2650	11124	47149	16061	24416
SLC31A1	3.9	27338	105362	99896	189796	91398
XPO7	3.8	5550	20916	14471	36507	30511
ITGA10	3.7	1643	6062	1747	31058	7992
TRIP13	3.4	834	2840	14533	7963	6929
PPAP2A	3.3	14292	47531	14347	275985	149722
MPZL2	3.3	7590	24992	4748	6021	19258
METTL7A	3.3	586	1915	3323	20452	14260
EMP3	3.2	175098	552127	134181	1438640	455855
KBTBD8	3.1	9346	29351	40664	34851	42462
VPS29	3.0	598	1809	10696	13180	13420
HLA-F	3.0	107	321	116	394	634
FZD2	3.0	394	1187	17485	56462	11380
TMEM30A	2.9	8242	24123	16591	39772	18198
PTPRE	2.9	1489	4343	6031	4579	7990
ANO6	2.8	64871	183765	78410	120341	94878
SLC9A6	2.8	2879	8031	4677	10122	3647
F11R	2.8	60498	168428	70872	15371	147765
ENTPD2	2.8	334207	928083	166011	43624	662929
RAB38	2.8	4258	11797	5205	517	6323
CSE1L	2.8	567064	1564572	1201389	1017165	1260009
SUSD2	2.7	19537	53666	17861	344487	834627
ARL6IP5	2.7	8558	23390	22150	87450	56160
SCAMP3	2.7	1710	4591	6312	6547	7444
NGFR	2.7	15882	42574	336188	25037	560759
YIPF6	2.7	3193	8478	20371	8827	16425
SLC5A6	2.6	32329	85279	226922	79071	90610

**SLC39A10**	2.6	4184	11022	26178	19459	21542
**DDR1**	2.6	8691	22891	62239	61605	128462
**CD9**	2.6	8766	23068	88463	63668	167647
**MCCC2**	2.6	14429	37911	6758	5805	7735
**THBD**	2.6	3973	10362	204832	9532	44312
**SIRPA**	2.6	198894	517087	191034	553094	464576
**SLC44A2**	2.6	133124	343949	420854	750214	720124
**YWHAG**	2.6	6860	17680	14967	64989	77326
**HLA-A**	2.6	201679	516403	128627	437352	576744
**ADGRE2**	2.5	66412	169044	61914	96608	293776
**FZD6**	2.5	6615	16634	16840	22937	36314
**YIPF5**	2.5	8960	22361	22558	29627	29822
**PROCR**	2.5	224389	556397	611397	774401	643747
**FAT2**	2.5	3225	7980	28625	15487	24598
**DFNA5**	2.5	1997	4936	8213	5726	8118
**TALDO1**	2.5	3369	8294	13716	13608	18193
**AGTRAP**	2.4	1682	4115	10658	3886	7112
**NME1**	2.4	52618	126847	144170	185933	160933
**EMB**	2.4	15023	36203	92733	44402	61355
**ATP5O**	2.4	50922	121908	16131	49369	31664
**PCCB**	2.4	207567	496645	247426	539209	450935
**TELO2**	2.4	3267	7735	1097	4177	2815
**NOTCH1**	2.4	8413	19801	38109	19590	36520
**CTPS1**	2.3	4244	9909	17216	6430	8033
**XPO5**	2.3	3508	8188	8408	232	288
**SLC7A5**	2.3	7382432	17196110	33473982	5057456	17599670
**ATP5B**	2.3	451641	1044555	411660	680103	493852
**CAP1**	2.3	23665	54563	104871	152229	90851
**ITGB4**	2.3	6402438	14745361	6806860	1632440	8009269
**TTYH3**	2.3	4542	10443	404	14943	2549
**TOMM22**	2.3	48809	111759	122695	67634	97767
**NRP1**	2.3	18739	42899	126308	1015839	151887
**TMEM165**	2.3	29441	67313	42657	66081	72454
**FAS**	2.3	99743	226480	310775	385534	515705
**PTK7**	2.3	604735	1370842	3017756	8767562	6893877
**SLC16A3**	2.3	34057	77189	121895	55133	51539
**SLC43A3**	2.3	170766	384365	367567	227402	376686
**PVR**	2.2	103779	232981	1056056	246189	366976
**CLDND1**	2.2	17700	39734	39698	60273	53902
**CD44**	2.2	5683399	12743353	27072292	21073190	16421299
**SLC9A1**	2.2	346548	776040	712108	612969	679011

**IPO9**	2.2	79155	177244	70451	128282	99049
**MCCC1**	2.2	58759	131246	67567	41677	76899
**EPHB4**	2.2	12185	27050	12336	66834	66571
**TNPO1**	2.2	89727	199041	172321	179401	131659
**ITGA6**	2.2	3243858	7163407	4790731	3151514	6492979
**ACACA**	2.2	1876997	4129162	1126181	442562	707621
**CA9**	2.2	798075	1743327	1218148	26162	420829
**ITGA3**	2.2	3959999	8640607	9890413	10599505	12180499
**SLC3A2**	2.2	51595	112249	257209	42871	149888
**XPO1**	2.2	165924	358990	252534	310341	252565
**CD276**	2.2	1231748	2664448	1341540	933030	1235261
**OTUB1**	2.2	11220	24122	72384	71486	76829
**STOML2**	2.1	27981	60135	44177	80845	72669
**MPZL1**	2.1	98441	211483	59819	232305	256733
**S100A14**	2.1	38195	81807	68350	3292	85429
**SLC1A5**	2.1	2626558	5614244	4726438	2799158	3860209
**IPO5**	2.1	228311	486979	378732	351264	292435
**SLC16A1**	2.1	3827	8161	28579	6750	12025
**TKT**	2.1	15150	32257	93370	91954	127740
**ABCC3**	2.1	39424	83738	17578	66548	62646
**NPTN**	2.1	976239	2067260	1206807	2207407	1325793
**PSMC1**	2.1	794	1678	3773	8357	2987
**MPDU1**	2.1	10899	23045	34255	44818	54175
**VAMP3**	2.1	5872	12402	38799	38639	38228
**HSPA8**	2.1	150089	315121	221296	167312	170240
**GNAI2**	2.1	86298	180618	77482	130073	85013
**MST1R**	2.1	156371	326325	236591	59545	260668
**VDAC1**	2.1	244678	508728	898194	989772	914283
**ENG**	2.1	7088	14692	37538	502	302
**ADGRE5**	2.1	264334	544050	182522	1362793	744713
**IPO7**	2.1	327593	674113	487523	360577	367422
**ITGB1**	2.1	9872795	20315012	35057319	32561027	28187248
**ATP1B3**	2.1	1447314	2975577	1652222	1584683	2621959
**TUBA1C**	2.1	132739	272278	96232	217990	184232
**SLC26A2**	2.0	69582	142081	107473	297853	130708
**IGF1R**	2.0	62119	126772	133807	60387	68568
**EPCAM**	2.0	106868	217948	403828	13208	213803
**SLC12A2**	2.0	65638	133769	199970	690374	258181
**GALNT2**	2.0	22823	46286	13275	42986	35680
**ATL3**	2.0	5642	11394	21210	55512	37737
**GBP2**	2.0	319	642	498	8742	4122

**SEC61A1**	2.0	75495	151870	218667	210921	208640
**CTNNA1**	2.0	1001655	2009974	970957	603979	863078
**SCARB1**	2.0	197596	396459	389180	46414	131235
**SLCO4A1**	2.0	16042	32058	384	0	754
**NOTCH2**	2.0	10998	21977	52890	81242	23973
**EGFR**	2.0	1028762	2052502	3180044	1735163	3203320

Supplemental Referencea

1. Zawel L, Dai JL, Buckhaults P, Zhou S, Kinzler KW, Vogelstein B, Kern SE. Human Smad3 and Smad4 are sequence-specific transcription activators. Molecular Cell. 1998; 1(4):611-617.

2. Rieger MA, Hoppe PS, Smejkal BM, Eitelhuber AC, Schroeder T. Hematopoietic cytokines can instruct lineage choice. Science. 2009; 325(5937):217-218. doi:10.1126/science.1171461.

3. Eilken HM, Nishikawa S-I, Schroeder T. Continuous single-cell imaging of blood generation from haemogenic endothelium. Nature. 2009; 457(7231):896-900. doi:10.1038/nature07760.

4. Yang J, Mani SA, Donaher JL, Ramaswamy S, Itzykson RA, Come C, Savagner P, Gitelman I, Richardson A, Weinberg RA. Twist, a master regulator of morphogenesis, plays an essential role in tumor metastasis. Cell. 2004; 117(7):927-939. doi:10.1016/j.cell.2004.06.006.

5. Nagarajan S, Hossan T, Alawi M, Najafova Z, Indenbirken D, Bedi U, Taipaleenmäki H, Ben-Batalla I, Scheller M, Loges S, Knapp S, Hesse E, Chiang CM, et al. Bromodomain protein BRD4 is required for estrogen receptor-dependent enhancer activation and gene transcription. Cell Rep. 2014; 8(2):460-469. doi:10.1016/j.celrep.2014.06.016.

6. Prenzel T, Begus-Nahrmann Y, Kramer F, Hennion M, Hsu C, Gorsler T, Hintermair C, Eick D, Kremmer E, Simons M, Beissbarth T, Johnsen SA. Estrogen-dependent gene transcription in human breast cancer cells relies upon proteasome-dependent monoubiquitination of histone H2B. Cancer Res. 2011; 71(17):5739-5753. doi:10.1158/0008-5472.CAN-11-1896.

7. Graessel A, Hauck SM, von Toerne C, Kloppmann E, Goldberg T, Koppensteiner H, Schindler M, Knapp B, Krause L, Dietz K, Schmidt-Weber CB, Suttner K. A combined omics approach to generate the surface atlas of human naive CD4+ T cells during early TCR activation. Mol Cell Proteomics. 2015; 14(8):2085-102. doi: 10.1074/mcp.M114.045690.

8. Grosche A, Hauser A, Lepper MF, Mayo R, von Toerne C, Merl-Pham J, Hauck SM. The Proteome of Native Adult Müller Glial Cells From Murine Retina. Mol Cell Proteomics. 2016; 15(2):462-480. doi:10.1074/mcp.M115.052183.

9. Hauck SM, Dietter J, Kramer RL, Hofmaier F, Zipplies JK, Amann B, Feuchtinger A, Deeg CA, Ueffing M. Deciphering membrane-associated molecular processes in target tissue of autoimmune uveitis by label-free quantitative mass spectrometry. Mol Cell Proteomics. 2010; 9(10):2292-2305. doi:10.1074/mcp.M110.001073.
